# Challenging, Accurate and Feasible: CAF-1 as a Tumour Proliferation Marker of Diagnostic and Prognostic Value

**DOI:** 10.3390/cancers13112575

**Published:** 2021-05-24

**Authors:** Alexandros G. Sykaras, Alexandros Pergaris, Stamatios Theocharis

**Affiliations:** 1 First Department of Pathology, Medical School, National and Kapodistrian University of Athens, 75 Mikras Asias Street, Bld 10, Goudi, 11527 Athens, Greece; alexander.sykaras@gmail.com (A.G.S.); alexperg@yahoo.com (A.P.); 2 Department of Pathology, Evangelismos General Hospital, Ipsilantou 45-47, 10676 Athens, Greece

**Keywords:** cancer, biomarker, proliferation, tumour, immunohistochemistry, grading, prognosis

## Abstract

**Simple Summary:**

There is an emerging need for new weapons in the battle against cancer; therefore, the discovery of new biomarkers with diagnostic, prognostic, and therapeutic value is a priority of current cancer research. An important task is to identify how quickly a tumour proliferates. A tumour’s proliferation rate is critical for grading and clinical decision-making; hence, there is an imperative need for accurate proliferation markers. Here, we review evidence demonstrating that chromatin assembly factor 1 (CAF-1) is a proliferation marker of clinical value. CAF-1 is selectively expressed in proliferating cells and its expression can be evaluated by immunohistochemistry in cytology smears and biopsies. CAF-1 expression is increased in almost all cancers and correlates strongly with the expression of Ki-67, the current routine proliferation marker. Overexpression of CAF-1 is associated with poor clinical outcome (advanced cancer stage, recurrence, metastasis, and decreased survival). CAF-1 is a robust, reproducible, and feasible proliferation marker of prognostic importance and may represent an attractive alternative or complementary to Ki-67 for cancer stratification and clinical guidance.

**Abstract:**

The discovery of novel biomarkers of diagnostic, prognostic, and therapeutic value is a major challenge of current cancer research. The assessment of tumour cell proliferative capacity is pivotal for grading and clinical decision-making, highlighting the importance of proliferation markers as diagnostic and prognostic tools. Currently, the immunohistochemical analysis of Ki-67 expression levels is routinely used in clinical settings to assess tumour proliferation. Inasmuch as the function of Ki-67 is not fully understood and its evaluation lacks standardization, there is interest in chromatin regulator proteins as alternative proliferation markers of clinical value. Here, we review recent evidence demonstrating that chromatin assembly factor 1 (CAF-1), a histone chaperone selectively expressed in cycling cells, is a proliferation marker of clinical value. CAF-1 expression, when evaluated by immunocytochemistry in breast cancer cytology smears and immunohistochemistry in cancer biopsies from several tissues, strongly correlates with the expression of Ki-67 and other proliferation markers. Notably, CAF-1 expression is upregulated in almost all cancers, and CAF-1 overexpression is significantly associated, in most cancer types, with high histological tumour grade, advanced stage, recurrence, metastasis, and decreased patient survival. These findings suggest that CAF-1 is a robust, reproducible, and feasible proliferation marker of prognostic importance. CAF-1 may represent an attractive alternative or complementary to Ki-67 for cancer stratification and clinical guidance.

## 1. Introduction

### Proliferation Markers as Prognostic Tools in Cancer

Cancer is the second leading cause of death worldwide, according to the World Health Organization (WHO) [1]. Despite recent progress in understanding the mechanisms of tumorigenesis, there is an imperative need for the discovery of novel biomarkers that can serve as diagnostic and prognostic tools. Uncontrolled cell proliferation is a hallmark of cancer development, and the expression of genes involved in cell proliferation is upregulated in tumours [2]. In parallel, the increased proliferative capacity of tumour cells is a characteristic of high-grade tumours and is associated with poor clinical outcome. Therefore, the precise assessment of cell proliferation remains critical for prognosis and therapeutic decisions [3], and the identification of reliable, robust, and feasible proliferation markers is a challenge of utmost importance. The immunohistochemical quantification of proliferation-associated protein expression in patient samples has been established as a surrogate index of tumour proliferation rate, together with mitotic index.

Immunohistochemical analysis of Ki-67 expression in tumour specimens is currently the most routinely used method to assess cancer cell proliferation in clinical pathology laboratories. Ki-67 is considered the archetypal proliferation marker, as it is expressed in all proliferative cells but, still, little is known about its biological function. Recent evidence suggests that Ki-67 is not critical for cell proliferation, but it is rather a regulator of chromatin organization [4] whose expression reflects the cell proliferation rate (albeit indirectly) [5]. Despite the fact that Κi-67 expression is used for grading purposes (as in neuroendocrine tumours) [6] and has been associated with aggressiveness, poor response to therapy, and decreased survival in several solid tumours [7,8], the inability to set universally accepted cut-off scores and evaluation guidelines cast doubt on its meaningfulness. Ki-67 immunoreactivity is extremely variable in pulmonary large cell neuroendocrine carcinomas and cannot be used as a diagnostic criterion or prognostic factor [9]. In breast cancer, the lack of reproducibility in Κi-67 labelling index scoring has limited its clinical use [10] and led to an extensive effort to develop an automated scoring system [11,12] and new standardization tools [13]. In ovarian cancer, Ki-67 is a useful stratification marker in early but not in advanced disease stage [14]. Additionally, it was recently suggested that Κi-67 is more of a graded than a binary marker, and its expression levels in cycling cells depends on how long they remained quiescent before entering the cell cycle. These data explain the variability of Ki-67 staining intensity in tumour specimens and prompts careful interpretation [15].

The use of other proliferation-associated proteins, such as proliferating cell nuclear antigen (PCNA) and mini chromosome maintenance proteins (MCM), as cancer proliferation biomarkers has been also suggested. PCNA is overexpressed in the S and G2 phases of cell cycle [16], but it is less sensitive and specific than Κi-67 and fails to correlate with clinicopathologic parameters [17]. On the other hand, MCM proteins are very sensitive markers of proliferation because they are expressed in both replication-competent and actively proliferating cells, staining a higher number of cells in tumour specimens than PCNA or Ki-67 [18]. MCM proteins show higher sensitivity and specificity than Ki-67 in some tumours, correlate with clinicopathologic parameters, and have diagnostic and prognostic value. However, it is not clear if the prognostic significance relies on the stand-alone expression of any of them or the expression pattern of all members of the MCM family [17,19,20,21,22].

Chromatin regulator proteins have been suggested as proliferation markers of potential clinical value. The expression of the chromatin regulator histone chaperone anti-silencing function 1b (ASF1b) protein is upregulated in breast cancer [23,24], correlates significantly with Κi-67 expression, and is an independent prognostic factor of disease progression and metastasis [23]. Similarly, heterochromatin protein isoform α (HP1α) is overexpressed in solid tumours and is an independent prognostic marker of reduced survival in breast cancer [25]. In prostate cancer, centromeric histone H3 variant centromere protein A (CENPA) mRNA expression correlates significantly with MKI67 gene expression. Additionally, increased levels of CENPA mRNA and protein are associated with aggressive biological behaviour (advanced stage and metastasis) [26]. In the luminal A breast cancer subtype, overexpression of the histone chaperone Holliday junction recognition protein (HJURP) is associated with aggressive behaviour and is an independent prognostic factor that outperforms Ki-67 [27].

The histone chaperone complex chromatin assembly factor 1 (CAF-1) is essential for life and has been studied extensively. In parallel with the molecular characterization of its function, CAF-1 has emerged as a challenging proliferation marker of cancer cells [28,29]. Here, we discuss the growing evidence that CAF-1 is a proliferation marker of diagnostic, prognostic, and predictive value and its expression correlates significantly with other established (Ki-67) or potential (MCMs) proliferation markers.

## 2. CAF-1 as a Proliferation Marker

**Figure 1:**
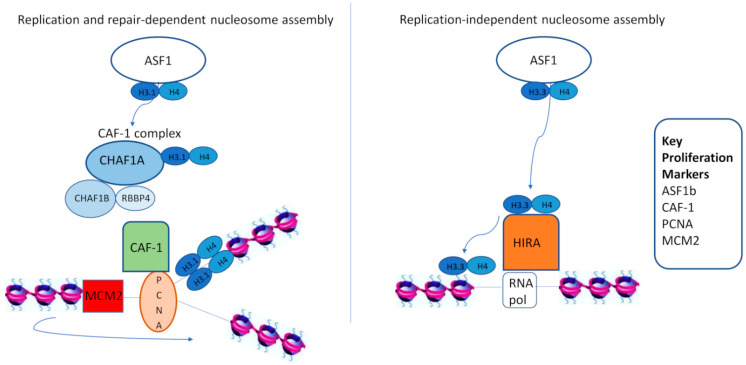
A simplified model of key histone chaperones and proliferation markers. CAF-1: chromatin assembly factor 1, HIRA: histone regulator A, PCNA: proliferating cell nuclear antigen, MCM: mini chromosome maintenance proteins, ASF1: anti-silencing function 1.

Histone chaperones are responsible for histone deposition on chromatin in two nucleosome assembly ways: one coupled to DNA synthesis, and one DNA-synthesis independent. Histone regulator A (HIRA) mediates histone variant deposition in a replication-independent nucleosome assembly pathway, whereas CAF-1 deposits histone variants in a DNA-synthesis coupled way [30]. CAF-1 is a heterotrimeric complex consisting of three subunits in human cells, encoded by the evolutionarily conserved genes CHAF1A (p150), CHAF1B (p60), and RBBP4 (p48). CAF-1 is expressed in cycling but not in quiescent cells, and has a pivotal role in chromatin organization during DNA replication, the maintenance of chromatin integrity, and the regulation of somatic cell identity [31,32,33,34,35]. Its primary role is to associate with newly synthesized histones (H3, H4, and particularly histone variant H3.1) and facilitate their deposition at the replication fork during S-phase [36,37]. The newly synthesized histones are delivered to the CAF-1 complex by ASF1, and their deposition on DNA is promoted via the interaction of CAF-1 with PCNA. Whereas CAF-1 deposits histone variants to newly synthesized DNA strands, MCM2 passes parental histones to them [38]. Apart from this role, CAF-1 is pivotal in chromatin repair after UV-induced DNA damage by incorporating the H3.1 variant to restore chromatin structure [36] (Figure 1).

In replication- or repair-dependent nucleosome assembly, the histone chaperone complex CAF-1 has a critical role in depositing newly-synthesized histone variant H3.1/H4 dimers (delivered to CAF-1 by ASF1) onto DNA strands. The action of CAF-1 is mediated via its interaction with PCNA. In parallel, MCM2 binds and passes parental histones to replicating DNA strands. In contrast with CAF-1, the HIRA complex incorporates Asf1-donated H3.3/H4 histones to non-nucleosomal DNA, a process facilitated by the interaction of HIRA with RNA polymerase II. Key histone chaperones that have been used as proliferation markers are highlighted in the inset box in Figure 1.

Almouzni and colleagues initially showed that the expression of CAF-1 subunits correlates with cell proliferation rate and is enhanced in tumour cells in comparison with normal tissues. Furthermore, they revealed that CHAF1B protein expression strongly correlates with Ki-67 expression in breast cancer cytology smears and biopsies (as shown in [28]) and is strongly associated with tumour size and grade, demonstrating that CAF-1 can be used as a proliferation marker of clinical importance [28]. A follow-up study from the same group confirmed the strong correlation of CHAF1B protein expression with Ki-67 labelling indices in breast, cervical, endometrial, renal, prostate, gastric, colon, pancreatic, and thyroid cancer (as shown in [29]). Additionally, CHAF1B levels were weakly but significantly associated with MCM (MCM2 and MCM5) expression in the same subset of tumours. Importantly, CHAF1B expression was associated with clinicopathologic data (tumour grade and staging) and was an independent prognostic factor for poor outcome in renal, endometrial, and cervical cancer [29].

## 3. CAF-1 Expression in Tumours: Correlation with Clinicopathologic Data and Survival

### CAF-1 Expression in Hematologic Malignancies

RNA sequencing data from the Broad Institute’s Cancer Cell Line Encyclopaedia (CCLE) show higher levels of CHAF1A (https://portals.broadinstitute.org/ccle/page?gene=CHAF1A) (accessed on 20 May 2021), CHAF1B (https://portals.broadinstitute.org/ccle/page?gene=CHAF1B) (accessed on 20 May 2021) and RBBP4 (https://portals.broadinstitute.org/ccle/page?gene=RBBP4) (accessed on 20 May 2021) expression in leukaemias and lymphomas in comparison to other tumours. Despite this pattern, there are very few studies investigating the expression of CAF-1 in hematologic malignancies. CHAF1B was shown to be upregulated in acute megakaryoblastic leukaemia cell lines derived from individuals with Down syndrome (DS-AMKL) [39]. Volk et al. showed that CHAF1B mRNA is overexpressed in human acute myeloid leukaemia (AML) [40]. They demonstrated that CHAF1B is required for haematopoiesis and is upregulated in human AML cell lines and patient-derived CD34+ cells. Importantly, this study provided a mechanistic link between CHAF1B overexpression and leukaemogenesis, showing that the CAF-1 mediated DNA replication-coupled nucleosome assembly competes with myeloid transcription factors and blocks the differentiation of leukaemic cells. This mechanism indicates that the CAF-1 complex may become a target for new therapeutic strategies [40,41]. Analysis of the AML dataset from The Cancer Genome Atlas (TCGA) revealed that there was no difference in CHAF1B mRNA levels between different subtypes of AML, and higher expression correlates with poor outcome [40]. In accordance with CCLE data, a transcriptomic analysis of another cohort identified the significant upregulation of RBBP4 mRNA in AML patients [42].

CAF-1 expression has not been studied in lymphoma patients. There is experimental evidence that Epstein–Barr virus (EBV), a virus associated with Burkitt and Hodgkin lymphoma, upregulates the expression of all CAF-1 subunits in newly-infected B cells and incorporates the CAF-1 nucleosome assembly mechanism to establish and maintain latency [43].

Recently, it was reported that CHAF1B protein is overexpressed in 27% of mycosis fungoides (MF) patient samples [44]. In this relatively small cohort of MF patients, CHA1B does not correlate with the stage of the disease, but is significantly associated with decreased overall survival (OS), representing a prognostic factor of poor outcome stronger than the stage of disease [44].

## 4. CAF-1 Expression in Solid Tumours

### 4.1. Gynaecologic Cancers

CHAF1A protein overexpression in cervical cancer is a poor prognostic factor that is associated with advanced stage, increased percentage of local recurrence, metastasis, and lower OS [45]. In cervical cancer cell lines, CHAF1A was shown to regulate cell proliferation, migration, and invasion, driving the essential biological processes for tumorigenesis and metastasis [45]. CHAF1B protein expression is higher in squamous than adenocarcinoma subtypes of cervical cancer [29]. Increased levels of CHAF1B correlate significantly with histological grade and reduced OS [29]. In contrast with the other CAF-1 subunits, the reduced expression of RBBP4 in cervical cancer modulates the transforming activity of HPV and promotes HPV16-associated carcinogenesis [46]. Similarly with cervical cancer, CHAF1B is a predictor of poor histological grade, advanced stage, and decreased OS in endometrial cancer [29]. Additionally, the CHAF1A protein is overexpressed in epithelial ovarian cancer (serous and mucinous), and higher expression is associated with advanced disease stage, spread to lymph nodes, and reduced OS [47]. Experiments in human ovarian cancer cell lines suggest that CHAF1B enhances the proliferation of cancer cells and inhibits apoptosis, thus mediating tumour growth [47].

### 4.2. Lung Cancer

CHAF1A mRNA and protein levels are higher in non-small cell lung carcinoma (NSCLC) tissues in comparison with normal lung parenchyma [48,49]. Overexpression of the CHAF1A protein is associated with tumour recurrence, metastatic spread, and poor outcomes (shorter OS and disease-free survival (DFS) rates) [49]. CHAF1A knock-down experiments in the H1299 NSCLC cell line indicate that CHAF1A promotes proliferation and inhibits apoptosis in cancer cells [48,49], effects that are blocked by the overexpression of miR-520b, which directly targets CHAF1A [50]. Notably, miR-520b levels in NSCLC tissue are lower than in normal lung [50].

Similar to CHAF1A, NSCLC tissues display increased CHAF1B mRNA and protein levels [51]. Higher expression levels of CHAF1B protein correlates significantly with male gender, smoking, squamous subtype, and poor differentiation of the tumour. Additionally, higher levels of CHAF1B are associated with increased tumour size, advanced stage, and decreased DFS and OS [51]. There is also a strong correlation between CHAF1B and Ki-67 expression in patient-derived tissue, indicating that CHAF1B may be used as a proliferation marker. Along this line, it has been shown that CHAF1B promotes the proliferation of NSCLC cells, both in the 95-D cell line and in a lung cancer xenograft mouse model, via inhibition of the p53-mediated apoptotic pathway [51].

### 4.3. Gastrointestinal Cancers

CHAF1A mRNA and protein expression are upregulated in colon cancer [52]. Overexpression of the CHAF1A protein is associated with advanced tumour stage, high-grade (poor differentiation) histology, and tumour invasion, and is an independent prognostic factor of poor clinical outcome (reduced OS and DFS) [52]. Functional experiments revealed that CHAF1A promotes proliferation and inhibits the apoptosis of colon cancer cells [52]. CHAF1B protein expression in colon cancer positively correlates with Ki-67 expression, but proved not to have prognostic value [29]. RBBP4 protein expression is upregulated in colon cancer cell lines and clinical samples [53,54]. RBBP4 promotes tumorigenesis via the activation of the Wnt/β-catenin pathway [54], and there is a significant association between RBBP4 expression and vascular invasion, lymph node spread, hepatic metastasis, and reduced survival [53].

CHAF1A mRNA and protein are also overexpressed in gastric cancer. The expression of the CHAF1A protein correlates with the percentage of Κi-67 positive cells and is associated with decreased OS [55]. Functional experiments suggest that CHAF1A may act as a co-activator of the Wnt pathway, interacting with TCF4 and increasing the expression of c-MYC and CCND1 to promote cancer cell proliferation. Interestingly, the same study provided a mechanistic link between *Helicobacter pylori* infection and CHAF1A overexpression, showing that *H. pylori* upregulates CHAF1A transcription via the Sp1 transcription factor [55]. CHAF1A expression is also associated with the survival of gastric cancer patients after radical gastrectomy and fluoropyrimidine-based (5-FU) adjuvant chemotherapy [56]. The overexpression of CHAF1A is an independent prognostic factor for poor response to 5-FU chemotherapy (reduced OS and DFS) in non-cardia gastric cancer. Higher expression of CHAF1A may upregulate the transcription of thymidylate synthetase (TS), a rate-limiting enzyme in folate metabolism responsible for 5-FU chemoresistance [56]. CHAF1B protein expression in gastric cancer correlates with the expression of the proliferative markers Ki-67, MCM2, and MCM5, but is not associated with clinical outcome or other clinicopathologic parameters [29].

Similar to tumours of the gastrointestinal tract, CHAF1A protein is overexpressed in hepatocellular carcinoma (HCC) and represents an independent prognostic factor indicative of reduced OS and DFS [57]. Additionally, CHAF1A expression correlated with increased tumour size, simultaneous multiple tumours, advanced stage, and higher Edmondson–Steiner histologic grade. CHAF1A promotes proliferation and inhibits the apoptosis of liver cancer cells in vitro, and induced HCC growth in a xenograft HCC mouse model [57]. Moreover, RBBP4 mRNA levels are upregulated in HCC specimens [58].

### 4.4. Urogenital Cancer

Immunohistochemistry of whole tissue sections and tissue microarrays revealed that CHAF1B protein expression is upregulated in prostate cancer. Higher expression of CHAF1B is associated with increased Κi-67 staining of cancer cells, and with aggressive clinical behaviour. CHAF1B levels correlate significantly with higher Gleason score, pathological stage, metastasis, and reduced OS [59,60].

CHAF1B protein expression is also a proliferation and prognostic marker for renal cell carcinoma, exhibiting a strong correlation with Ki-67 levels and significant associations with poor differentiation of tumour cells, age, advanced stage, and lower OS [29].

### 4.5. Nervous System Tumours

CHAF1A protein overexpression in glioblastoma correlates with increased tumour size, advanced grade (III or IV according to WHO classification), and poor OS [61]. In vitro data suggest that high expression of CHAF1A induces tumour cell proliferation and inhibits apoptosis via the Akt-mediated activation of the Foxo3a/Bim signalling pathway [61]. Additionally, high levels of CHAF1B mRNA and protein indicate increased proliferation, correlate strongly with Ki-67 percentage [62], and are associated with advanced grade (grade IV) and decreased OS [62,63].

The overexpression of CHAF1A protein in neuroblastoma contributes to increased proliferation and tumour growth, and is an independent prognostic factor for advanced stage, and reduced DFS and OS [64,65]. Higher expression of CHAF1A correlates with a more aggressive phenotype characterized by undifferentiated morphology, and may induce metabolic pathways that contribute to the Warburg effect [65]. RBBP4 interacts with armadillo repeat-containing 12 (ARMC2) to mediate the repression of tumour suppressor genes in neuroblastoma [66]. RBBP4 protein levels are associated with poor differentiation, higher mitotic index, advanced staging, and reduced OS [66].

### 4.6. Breast Cancer

CHAF1A protein expression in breast cancer correlates with Ki-67 expression, poor histological grade, high mitotic index and decreased DFS [23,27]. CHAF1B expression also correlates with Ki-67 percentage [27,28,29] and is associated with increased tumour size [23,28], poor histological grade [23,28,29], and high mitotic index [27,28]. Additionally, it had been suggested that CHAF1B is an independent prognostic factor for decreased DFS and OS [23], although this association was not confirmed in other cohorts [27,29].

In contrast with Ki-67, CAF-1 mRNA levels can be used to distinguish the molecular subtypes of breast cancer. CHAF1B mRNA expression is associated with negative oestrogen-receptor status [28] and is significantly lower in the luminal A breast cancer subtype in comparison with luminal B, HER2-positive, and basal-like subtype (BLC) tumours [27]. CHAF1A expression is upregulated in luminal B and basal-like tumours in comparison to luminal A tumours, but does not differ between luminal A and HER2-positive tumours [27]. Recently, it was shown that RBBP4 and HDAC1 proteins are overexpressed in breast cancer [67]. RBBP4 expression is higher in triple-negative and HER2-positive tumours in comparison with luminal A and luminal B tumours, correlates positively with HDAC1 expression, and correlates negatively with ER and PR expression. Moreover, high expression of the RBBP4 protein correlates significantly with lymph node metastasis and decreased survival [67].

### 4.7. Skin Cancer

The CHAF1B protein is upregulated in malignant melanoma (MM), particularly in the malignant melanocytes of the vertical growth phase. CHAF1B expression levels correlate significantly with increased Breslow depth, advanced staging, recurrence, and metastatic spread (skin, nodal, or distant), and is a predictor of poor survival [68]. A subset of MM is characterized by the overexpression of CHAF1B and poly (ADP-ribose) polymerase 1 (PARP-1), another regulator of DNA repair that is associated with aggressive melanoma behaviour [69]. In these MMs, CHAF1B expression is the strongest stand-alone predictor of nodal and distant metastases [70]. Similarly to MM, CHAF1B protein expression is upregulated in skin squamous cell carcinoma (SCC) and correlates with aggressive behaviour (relapse, metastasis, and decreased OS) [59].

### 4.8. Head and Neck Cancers

The RBBP4 protein is overexpressed in papillary, follicular, and anaplastic thyroid carcinoma, with the highest expression in anaplastic malignancy. Increased levels of RBBP4 promote tumour growth and mirror the increased nuclear factor κB (NFκΒ) activity in these tumours [71].

The CHAF1B protein is overexpressed in both benign and malignant salivary gland tumours in comparison to normal salivary glands. Importantly, CHAF1B and Ki67 expression can be used as markers of tumour aggressiveness, and CHAF1B expression levels are the best indicator of benign tumours that trans-differentiate to a malignant phenotype. Similarly, CHAF1B expression in malignant SGT is associated with tumour recurrence and represents the best prognostic factor for distant metastasis. Therefore, CHAF1B expression in SGT may represent an overall hallmark for aggressive biological behaviour [72].

CHAF1B is overexpressed in SCC of the tongue. CHAF1B protein expression correlates with PCNA levels and, in contrast with PCNA, is significantly associated with advanced stage, local recurrence, distant metastasis, and reduced overall survival [73]. In contrast with CHAF1B, CHAF1A protein expression inversely correlates with PCNA levels and is downregulated in aggressive tumours [73]. The overexpression of CHAF1B and abolished expression of CHAF1A are prognostic factors that identify oral SCC with aggressive biological behaviour. A subset of oral SCC with CHAF1A-low/CHAF1b-high expression has the worst prognosis, especially for middle-aged patients [73]. The overexpression of CHAF1B in oral SCC is restricted to HPV-negative tumours, whereas HPV-positive tumours display minimal CHAF1B expression [74]. HPV-negative oral SCC with high levels of CHAF1B, PARP-1, and nestin expression have increased metastatic potential and poor clinical outcome [74]. In vitro experiments suggest that CAF-1 depletion in these aggressive tumours induces sensitivity to PARP inhibitors and ionizing radiation, highlighting CAF-1 as a potential pharmacological target [75].

**Figure 2:**
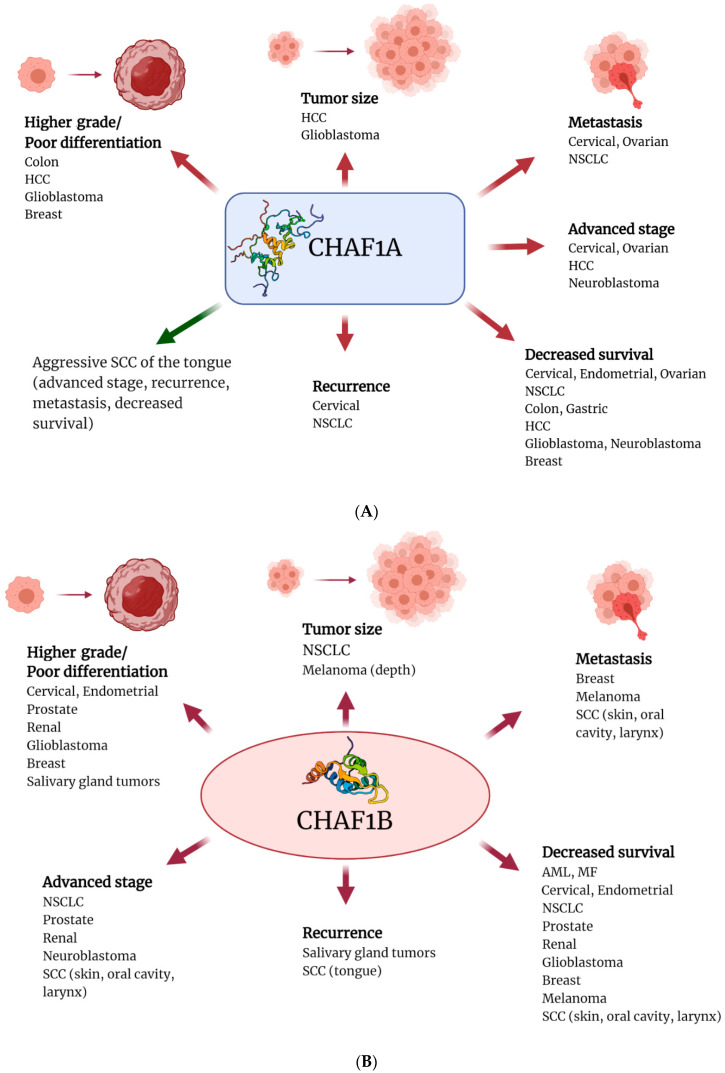

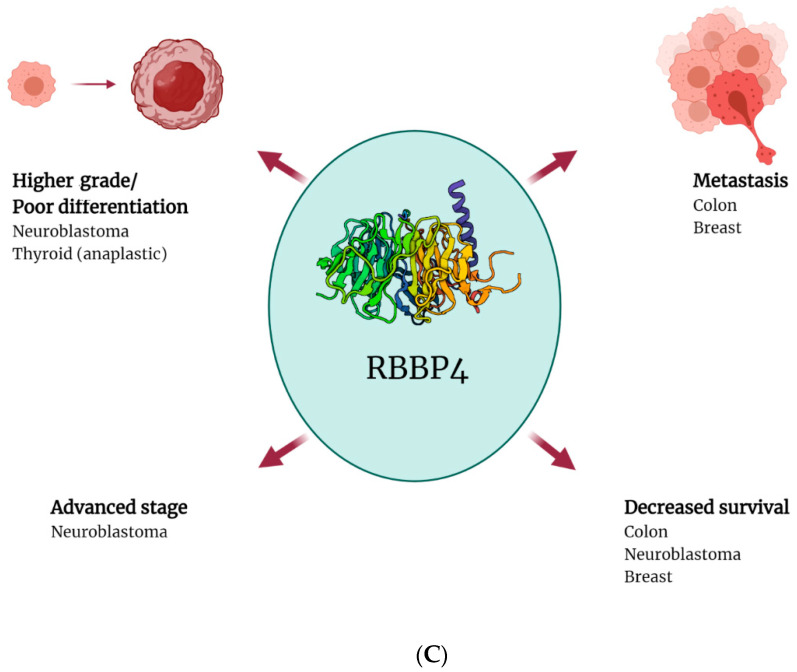
Correlation of CAF-1 expression with clinicopathologic parameters and survival. (**A**,**B**) CHAF1A and CHAF1B overexpression (red arrows) are associated with increased tumour size, higher grade, advanced stage, recurrence, metastasis, and reduced survival in tumours. Low expression of CHAF1A (green arrow) is associated with the aggressive biological behaviour of SCC of the tongue. (**C**) RBBP4 overexpression correlates with high grade, advanced stage, metastasis, and reduced survival in a subset of human cancers. This figure was created with BioRender.com. AML: acute myeloid leukaemia, MF: mycosis fungoides, NSCLC: non-small cell lung cancer, SCC: squamous cell carcinoma, HCC: hepatocellular carcinoma.

The correlation of CAF-1 expression with other clinicopathologic parameters and survival in tumours is summarized in Table 1 and Figure 2. The correlation of CAF-1 expression with Ki67, MCM, PCNA, and ASF1b expression in tumours is summarized in Table 2.

## 5. Mechanistic Insights-Targeting CAF-1

*In vitro* and *ex vivo* experiments suggest that CAF-1 overexpression inhibits apoptosis and promotes tumour growth. Even though the biological role of the CAF-1 complex is well studied, the mechanistic link between CAF-1 and tumorigenesis remains unclear. An outstanding question about the role of other histone chaperones in this process remains to be answered. Epigenomic and transcriptional reprogramming occurs during carcinogenesis, and histone chaperons play a critical role in these dynamic processes. Recently, it was suggested that CAF-1 downregulation may paradoxically promote epithelial to mesenchymal transition and the metastatic spread of breast cancer, a process regulated by other histone chaperones [76]. The identification of cancer types driven by CAF-1 and the elucidation of the molecular mechanisms linking CAF-1 with tumorigenesis may lead to the development of new therapeutic tools targeting CAF-1 in selected subsets of cancer patients.

## 6. Conclusions

CAF-1 (in particular CHAF1B) expression in a wide range of tumours displays a strong correlation with the prototype cell proliferation marker Ki-67 and other proliferation markers (PCNA, MCMs, and ASF1b). Importantly, CAF-1 expression is significantly associated with several clinicopathologic parameters such as tumour grade, stage, recurrence, metastasis, and survival. This finding is reproducible in different cohorts of breast cancer patients, regardless of the method used for CAF-1 assessment (immunocytochemistry in cytology samples or immunohistochemistry in biopsies). These data demonstrate that the immunohistochemical evaluation of CAF-1 expression is a reproducible and feasible tool to assess tumour proliferation, even in cytology samples. The accumulating evidence that the expression of CAF-1 subunits (especially CHAF1B and CHAF1A) has diagnostic and prognostic value advocates for the consideration of CAF-1 as a useful proliferation marker in the clinic. Can CAF-1 be used as a stratification marker in human tumours? To answer this key question, we need more studies that aim to elucidate the expression pattern of CAF-1 subunits and their prognostic significance in different cancer types.

## Figures and Tables

**Table 1:** Correlation of CAF-1 expression with clinicopathologic parameters and survival in human cancer. Red denotes overexpression, blue denotes reduced expression.

Tumour	CAF-1 Subunit	Clinicopathologic Parameters	Survival	Reference
AML	*CHAF1B* mRNA	-	↓ OS	[39,40]
	*RBBP4* mRNA	-	-	[42]
MF	CHAF1B	-	↓ OS	[44]
Cervical cancer	CHAF1A	Advanced stage Local recurrence Distant metastasis	↓ OS	[45]
	CHAF1B	Histological grade	↓ OS	[29]
	RBBP4	-	-	[46]
Endometrial cancer	CHAF1B	Histologic grade Advanced stage	↓ OS	[29]
Ovarian cancer	CHAF1A	Advanced stage Lymph node metastasis	↓ OS	[47]
NSCLC	*CHAF1A* (mRNA) CHAF1A (protein)	- Local recurrence Metastasis	- ↓ OS and DFS	[48] [49]
	CHAF1B	Male gender Smokers Squamous subtype Increased tumour size Advanced stage	↓ OS and DFS	[51]
Colon cancer	CHAF1A	Advanced stage Tumour invasion Poor differentiation	↓ OS and DFS	[52]
	CHAF1B	No correlation	-	[29]
	RBBP4	Vascular invasion Lymph node spread Hepatic metastasis	↓ OS	[53]
Gastric cancer	CHAF1A	-	↓ OS	[55]
	CHAF1A	Resistance to 5-FU chemotherapy (↓ OS and DFS)		[56]
	CHAF1B	No correlation	-	[29]
Hepatocellular carcinoma	CHAF1A	Increased tumour size Multifocal tumours Advanced stage Advanced Edmondson–Steiner grade	↓ OS and DFS	[57]
	*RBBP4* (mRNA)	-	-	[58]
Prostate cancer	CHAF1B	Higher Gleason score Advanced stage	↓ OS	[59,60]
Renal cancer	CHAF1B	Age Histological grade Advanced stage	↓ OS	[29]
Glioblastoma	CHAF1A	Increased tumour size Advanced grade	↓ OS	[61]
	CHAF1B	Advanced grade	↓ OS	[62]
Neuroblastoma	CHAF1A	Advanced stage	↓ OS and DFS	[65]
	RBBP4	Poor differentiation High mitotic index Advanced staging	↓ OS	[66]
Breast cancer	CHAF1A	High mitotic index Poor histological grade	↓ DFS	[23,27]
	CHAF1B	Increased tumour size Poor histological grade High mitotic index	↓ OS and DFS [23]	[23,28,29] [23,27]
	RBBP4	Lymph node metastasis	↓ OS	[67]
Melanoma	CHAF1B	Breslow depth Metastasis	↓ OS and DFS	[68,70]
Skin SCC	CHAF1B	Recurrence Metastasis	↓ OS	[59]
Thyroid cancer	RBBP4	Poor (anaplastic) morphology	-	[71]
Salivary gland tumours	CHAF1B	Malignant potential of benign tumours Recurrence Metastasis	-	[59,72]
SCC of the tongue	CHAF1A	Advanced stage Recurrence Metastasis	↓ OS	[73]
SCC of the oral cavity	CHAF1B	Advanced stage Recurrence Metastasis	↓ OS	[73,74,75]
SCC of the larynx	CHAF1B	Advanced stage Recurrence Metastasis	↓ OS	[59]

AML: acute myeloid leukaemia, MF: mycosis fungoides, NSCLC: non-small cell lung cancer, SCC: squamous cell carcinoma, OS: overall survival, DFS: disease-free survival.

**Table 2:** Correlation of CAF-1 expression with the expression of other proliferation markers in human cancers. Statistical data were retrieved from the referenced articles.

Tumour	CAF-1 Subunit	Marker	Significance	Reference
Breast	CHAF1B	Ki-67	r = 0.97; *p* < 10^−4^	[29]
Breast (smear)	CHAF1B	Ki-67	r = 0.94; *p* < 10^−4^	[28]
Breast (smear)	CHAF1B	PCNA	r = 0.95; *p* = 10^−4^	[28]
	CHAF1A	ASF1B	r = 0.6; *p* < 10^−10^	[23]
	CHAF1B	ASF1B	r = 0.7; *p* < 10^−9^	[23]
	CHAF1A	Ki-67	r = 0.69; *p* < 10^−3^	[27]
	CHAF1B	Ki-67	r = 0.65; *p* < 10^−3^	[27]
Endometrial	CHAF1B	Ki-67	r = 0.98; *p* < 10^−4^	[29]
Cervical	CHAF1B	Ki-67	r = 0.80; *p* < 10^−4^	[29]
Prostate	CHAF1B	Ki-67	r = 0.95; *p* < 10^−4^	[29]
Colon	CHAF1B	Ki-67	r = 0.97; *p* < 10^−4^	[29]
Gastric	CHAF1B	Ki-67	r = 0.89; *p* < 10^−4^	[29]
	CHAF1B	MCM2	r = 0.64; *p* < 10^−4^	[28]
	CHAF1B	MCM5	r = 0.49; *p* < 10^−4^	[28]
Pancreatic	CHAF1B	Ki-67	r = 0.99; *p* < 10^−4^	[29]
Renal	CHAF1B	Ki-67	r = 0.96; *p* < 10^−4^	[29]
Thyroid	CHAF1B	Ki-67	r = 0.86; *p* < 10^−4^	[29]
NSCLC	CHAF1B	Ki-67	r = 0.86; *p* < 10^−4^	[51]
SCC (tongue)	CHAF1A	PCNA	Inverse correlation	[73]
	CHAF1B	PCNA	Correlation	[73]

r: Spearman’s rank correlation coefficient, NSCLC: non-small cell lung cancer, SCC: squamous cell carcinoma, PCNA: proliferating cell nuclear antigen, MCM: mini chromosome maintenance proteins, ASF1b: anti-silencing function 1b.

## Data Availability

The data presented in this study are available on request from the corresponding author.
